# Serum Hepatitis B Surface Antigen Levels and Their Utility as a Predictor of Sustained Virological Response After Antiviral Treatment

**DOI:** 10.5812/hepatmon.6201

**Published:** 2012-07-30

**Authors:** Peter Karayiannis

**Affiliations:** 1 Hepatology and Gastroenterology Section Department of Medicine, Imperial College London, London, England

**Keywords:** Serum, Hepatitis B, Hepatitis B Surface Antigens

The hepatitis B surface antigen (HBsAg), first named as Australia antigen, was identified and linked to hepatitis in the late 1960s by Blumberg and colleagues. It is now well established that this protein forms the outer envelope of the hepatitis B virus (HBV) and constitutes the main diagnostic marker of both acute and chronic infection. This is facilitated by the fact that the protein is produced in large amount, not all of which is virus associated. Apart from the mature infectious virions known as Dane particles, two types of subviral particles devoid of nucleic acid are also produced; the 22nm spheres and filaments of longer length. The latter two forms outnumber virions by as much as 105. The high levels of circulating HBsAg have been linked to potential T-cell anergy during chronic infection, thus constituting a mechanism of immune evasion. There are 3 forms of the protein known as the small (S), medium (M) and Large (L), the latter two of which contain the S domain at their carboxyl end which is preceded by the Pre-S2 domain in the case of the M protein and Pre-S1+Pre-S2 domains in the L protein. These proteins are translated from the relevant mRNAs which are transcribed from the intrahepatic covalently closed circular DNA (cccDNA), and their level may thus reflect the replicative capacity of the virus. However, HBsAg may also be encoded by HBV-DNA sequences integrated into the hepatocyte DNA [1]. The efficacy of antiviral drugs such as pegylated interferon alpha (Peg-IFNa) or nucleos(t)ide analogues (NUCs) which are used in the treatment of chronic HBV carriers is monitored by measurement of HBV-DNA levels by sensitive real time polymerase chain reaction techniques. In recent years, quantitative measurement of HBsAg levels expressed in IU/ml has become possible through the use of two commercially available tests, namely the Architect QT and the Elecsys HBsAg II Quant assays. A number of studies have attempted to correlate HBsAg levels with the various phases of the natural course of chronic HBV infection, as well as determine whether they can be used as a predictive factor for a favourable outcome following antiviral treatment. In the former case, HBsAg levels during the immune tolerant phase appear to have a mean of 4.75 log10 IU/ml, as opposed to 4.2, 2.7 and 3.5 log10 IU/ml in the immune clearance, immune control and reactivation phases respectively. HBV DNA levels appear to mirror the HBsAg changes during the various phases, except for the immune control phase where HBV-DNA may be detectable at very low levels or not at all [2][3]. There is evidence to suggest that HBsAg levels may differ depending on genotype, these being higher in patients infected with genotype A and lowest in genotype D, with those for genotypes B and C being intermediate in a transfection system [4]. However, using patient sera in the immune clearance phase, Sonneveld and colleagues reported higher levels of HBsAg for genotypes A and D than B and C [5]. Clearly, this issue warrants further thorough investigation with clear definition of the patients’ stage of chronic liver disease. Various investigators have attempted to use HBsAg and HBV-DNA levels as a guide in defining a cut-off value for separating inactive carriers from those with active disease. HBsAg and HBV-DNA levels of < 1000 and ≤ 2000 IU/ml in HBeAg-ve patients have been shown to identify patients with inactive disease with a positive predictive value of 87.9% [3][5][6]. What is more, it has been suggested that HBsAg and HBV DNA levels of < 100 and < 200 IU/ml respectively, can predict HBsAg loss within 6 years with a diagnostic accuracy of 91.5%, sensitivity of 83.3%, specificity of 92.1%, positive predictive value of 45.5%, and negative predictive value of 98.6% [7].

HBsAg levels decline with Peg-IFN treatment in both HBeAg+ and HBeAg- patients. In HBeAg+ patients, this decline from baseline to week 48 averages 0.75 log10 IU/ml and there appears to be an additive effect when used in combination with a NUC (Lamivudine, Telbivudine) leading to a drop of 1.3 log10 IU/ml [8][9]. In the case of HBeAg- patients the drops are still substantial, but there is no difference when Peg-IFN is used in combination with a NUC (0.65 log10 IU/ml) [10]. When NUCs are used alone a more modest drop in HBsAg level is recorded ranging from 0.1-0.4 log10 IU/ml [9][10][11]. Quantitative measurements of HBsAg levels at 12 and 24 weeks of treatment with Peg-IFN in HBeAg+ patients have shown that a no decline in HBsAg or levels > 20000 IU/ml are associated with poor sustained virological response (SVR) ranging from 3-18% at both time points [8], whilst a decline to 1500 IU/ml is associated with SVR in 54-58% of patients [12][13], rising to 62-75% if the levels drop to < 300 IU/ml [14]. Similarly, HBeAg- patients with a decline in HBsAg levels by10% or more had a 47% and 43% chance of reduced HBV-DNA levels to < 4 log10 at 1 year depending on whether treatment response could be predicted by measurements at 12 or 24 weeks respectively. Moreover, such patients had a 22.5% chance of losing HBsAg at 5 years, and this increased to 30% if at 48 weeks HBsAg levels had dropped by > 1 log10 [15][16]. The utility of measuring HBsAg levels during NUC treatment is less clear as can be deduced from the small number of studies published, which appear to have included in the analysis heterogeneous groups of patients. The decline in HBsAg levels is slower and does not correlate with HBV-DNA levels. However, a rapid decline in HBsAg by >1 log10 IU/ml during therapy with Entecavir was associated with a higher loss of HBeAg after 1 year of treatment [11]. HBsAg seroclearance in HBeAg+ patients treated either with Tenofovir, Entecavir or Lamivudine ranged between 2-8% after 1-3 years of treatment, whilst in the case of HBeAg- patients, HBsAg loss occured less frequently and estimates suggest that this may require periods longer than 10 years [17][18].

In conclusion, a reduction in HBsAg levels during treatment, particularly at 12 and 24 weeks, may be predictive of a favourable outcome in the case of Peg-IFN treatment in HBeAg+ positive patients. However, suitable cut-off levels need to be more accurately defined. In the case of HBeAg- patients, both HBsAg and HBV-DNA decline levels at 12 weeks can be used as a guide to applying stopping rules. The picture in patients treated with NUCs is far from clear at the moment and clarification of the issues involved must await further detailed studies.
